# Significance of linkage disequilibrium and epistasis on genetic variances in noninbred and inbred populations

**DOI:** 10.1186/s12864-022-08335-9

**Published:** 2022-04-09

**Authors:** José Marcelo Soriano Viana, Antonio Augusto Franco Garcia

**Affiliations:** 1 Department of General Biology, Federal University of Viçosa, Viçosa, MG 36570-900 Brazil; 2 Department of Genetics, Luiz de Queiroz College of Agriculture, University of São Paulo, Piracicaba, SP 13418-900 Brazil

**Keywords:** Linkage disequilibrium, Epistasis, Inbreeding, Genetic variances

## Abstract

**Background:**

The influence of linkage disequilibrium (LD), epistasis, and inbreeding on genotypic variance continues to be an important area of investigation in genetics and evolution. Although the current knowledge about biological pathways and gene networks indicates that epistasis is important in determining quantitative traits, the empirical evidence for a range of species and traits is that the genotypic variance is most additive. This has been confirmed by some recent theoretical studies. However, because these investigations assumed linkage equilibrium, considered only additive effects, or used simplified assumptions for two- and higher-order epistatic effects, the objective of this investigation was to provide additional information about the impact of LD and epistasis on genetic variances in noninbred and inbred populations, using a simulated dataset.

**Results:**

In general, the most important component of the genotypic variance was additive variance. Because of positive LD values, after 10 generations of random crosses there was generally a decrease in all genetic variances and covariances, especially the nonepistatic variances. Thus, the epistatic variance/genotypic variance ratio is inversely proportional to the LD level. Increasing inbreeding increased the magnitude of the additive, additive x additive, additive x dominance, and dominance x additive variances, and decreased the dominance and dominance x dominance variances. Except for duplicate epistasis with 100% interacting genes, the epistatic variance/genotypic variance ratio was proportional to the inbreeding level. In general, the additive x additive variance was the most important component of the epistatic variance. Concerning the genetic covariances, in general, they showed lower magnitudes relative to the genetic variances and positive and negative signs. The epistatic variance/genotypic variance ratio was maximized under duplicate and dominant epistasis and minimized assuming recessive and complementary epistasis. Increasing the percentage of epistatic genes from 30 to 100% increased the epistatic variance/genotypic variance ratio by a rate of 1.3 to 12.6, especially in inbred populations. The epistatic variance/genotypic variance ratio was maximized in the noninbred and inbred populations with intermediate LD and an average allelic frequency of the dominant genes of 0.3 and in the noninbred and inbred populations with low LD and an average allelic frequency of 0.5.

**Conclusions:**

Additive variance is in general the most important component of genotypic variance. LD and inbreeding have a significant effect on the magnitude of the genetic variances and covariances. In general, the additive x additive variance is the most important component of epistatic variance. The maximization of the epistatic variance/genotypic variance ratio depends on the LD level, degree of inbreeding, epistasis type, percentage of interacting genes, and average allelic frequency.

**Supplementary Information:**

The online version contains supplementary material available at 10.1186/s12864-022-08335-9.

## Background

Basic knowledge on the genetics of quantitative traits was provided by RA Fisher [1], including the partitioning of the genotypic value in effects due to individual genes, allelic interactions (dominance), and nonallelic interaction (epistasis). Furthermore, he also recognized the significance of the linkage phase between genes on the population variance and on the correlation between relatives. The influence of linkage disequilibrium (LD), epistasis, and inbreeding on genotypic variance continues to be an important area of investigation in genetics and evolution [2–4]. Assuming linkage equilibrium, multilocus model, and three to five loci interactions, A Maki-Tanila and WG Hill [4] concluded that most genotypic variance is additive, regardless of the order of interaction, allelic frequencies, and type and magnitude of interaction effects. Another main finding was that the majority of the epistatic variance is due to digenic interactions. Assuming LD and a two- to three-locus model, WG Hill and A Maki-Tanila [3] showed that variances are generally higher with positive LD and that the epistatic variance/genotypic variance ratio is largest with negative LD. Both studies showed that epistatic variance is increased by increasing heterozygosity. However, this has no impact on the relative magnitude of the epistatic variance because the additive and epistatic variances increase in similar proportions.

Based on the additive model, J Clo, J Ronfort and D Abu Awad [2] showed that assuming stabilizing selection and high mutation rates, self-pollinated populations are able to accumulate genetic variation through negative LD. Using a meta-analysis of quantitative trait heritability, J Clo, L Gay and J Ronfort [5] confirmed previous theoretical and empirical evidence that self-pollinated populations exhibit lower levels of additive variance for quantitative traits. However, the decrease in the additive variance is compensated by the nonadditive components of genotypic variance. Because of negative consequences (inbreeding depression), geneticists agree that inbreeding should be efficiently controlled to maintain adequate genetic diversity in populations [6, 7]. However, self-pollination has been deliberately used in maize hybrid breeding (currently to a lesser extent due to doubled-haploid technology). For self-pollinated crops, the development of varieties involves selection over generations of increasing inbreeding. In these populations, inbreeding has an impact on the genetic variances and covariance between relatives [8].

Although the current knowledge about biological pathways and gene networks implies that epistasis is important in determining quantitative traits, the empirical evidence for a range of species and traits indicates that genotypic variance is most additive [9, 10]. Based on theoretical models, WG Hill, ME Goddard and PM Visscher [10] concluded that this occurs because of high differences in allelic frequencies. They also concluded that in outbred populations, the detection of epistasis is difficult unless the epistatic effects are large and the allelic frequencies are intermediate. TFC Mackay [9] emphasized that because epistasis regularly determines quantitative traits, it has consequences for plant and animal breeding, evolutionary biology, and human genetics. Recent studies on genomic selection and GWAS, including epistasis, have confirmed that most genetic variance is additive [11–14]. However, incomplete LD at low marker density can indicate epistasis when trait determination is purely additive [15].

The most important quantitative genetics theory for modeling epistasis was developed by O Kempthorne [16]. CC Cockerham [17] also provided a significant contribution. If modeling only inbreeding, LD, or epistasis is a difficult task for quantitative geneticists, jointly modeling the three events is a challenge. An impressive approach for two-genes theory in quantitative genetics assuming inbreeding, LD, and epistasis was presented by BS Weir and CC Cockerham [18]. Because of the complexity of the expressions for the genetic variances and covariance between relatives, they concluded that “the result is of little use”. That is, the functions do not allow assessing the influence of LD, epistasis, and inbreeding on the genetic variability and the degree of relationship in the populations. Furthermore, because recent investigations based on theoretical models assumed linkage equilibrium, considered only additive effects, or used simplified assumptions for two- and higher-order epistatic effects, the objective of this study was to provide additional information about the impact of LD and epistasis on the genetic variances in noninbred and inbred populations, using a simulated dataset.

## Results

The analysis of the parametric LD in the populations shows that the LD level depends mainly on the gene density (Additional Fig. 1). A higher LD level was observed under high gene density (one gene/cM). Regardless of the gene density, the LD level was generally higher for the closest genes. Because the LD was positive, 10 generations of random crosses significantly decreased the LD level of the populations. The decrease was higher for the density of one gene/5 cM, regardless of the population (approximately − 95% for r^2^, on average). The average decrease in r^2^ for the density of one gene/cM was − 81%. The LD level showed only a slight decrease after 10 generations of selfing regardless of the population (approximately − 14% for r^2^, on average).

**Fig. 1:**
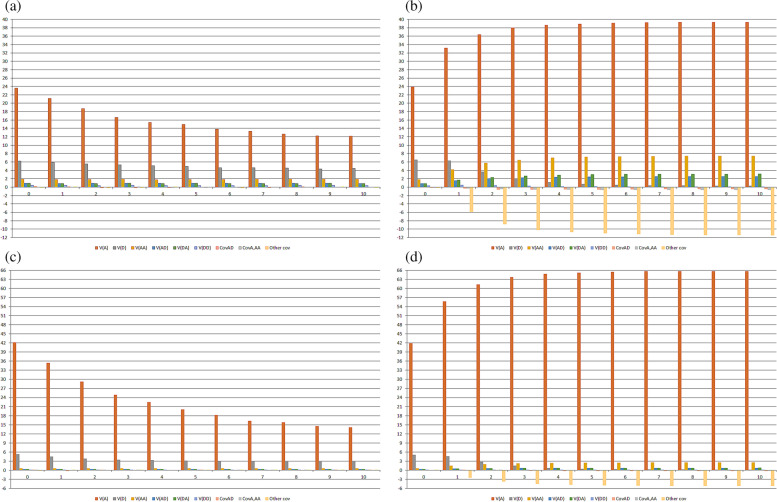
Components of the genotypic variance in population with high LD level, along 10 generations of random crosses (**a** and **c**) or selfing (**b** and **d**), assuming an admixture of digenic epistasis, 100 (**a** and **b**) and 30% (**c** and **d**) epistatic genes, and sample size of 5000 per generation

To assess the significance of LD and epistasis on the magnitude of the genotypic variance components, we assumed the density of one gene/5 cM. In general, regardless of the type of epistasis, percentage of interacting genes, LD level, and degree of inbreeding, the most important component of the genotypic variance was the additive variance (Additional Figs. 2 to 8 and Fig. 1). Only under duplicate epistasis, 100% epistatic genes, and F ≥ 7/8 was the additive x additive variance higher than the additive variance (Additional Fig. 3). The impact of LD on the genetic variances and covariances is shown by the changes in their magnitudes over the random cross generations (Additional Figs. 2 to 8 and Fig. 1). Because of positive LD values, after 10 generations of random crosses there was generally a decrease in all genetic variances and covariances, especially the nonepistatic variances. The decreases in the additive and dominance variances ranged between − 28 and − 70% and − 12 to − 62%, respectively, depending on the type of epistasis and the percentage of interacting genes. The changes in the epistatic variances were much lower, ranging from 0.5 to − 13%. Thus, the epistatic variance/genotypic variance ratio is inversely proportional to the LD level.

Because there was only a slight decrease in the LD level with inbreeding, the changes in the magnitudes of the genetic variances and covariances over generations of selfing are mainly attributable to inbreeding. Increasing inbreeding increased the magnitude of the additive,

additive x additive, additive x dominance, and dominance x additive variances, and decreased the dominance and dominance x dominance variances (Additional Figs. 2 to 8 and Fig. 1). The additive variance increased from 50 to 76% and the epistatic variances increased in the range 114 to 863%, depending on the type of epistasis and the percentage of epistatic genes. The decreases in the dominance and dominance x dominance variances were similar, in the range -76 to -98%. Except for duplicate epistasis with 100% interacting genes, the epistatic variance/genotypic variance ratio was proportional to the level of inbreeding.

In general, additive x additive was the most important component of epistatic variance, regardless of the type of epistasis, percentage of epistatic genes, LD level, and degree of inbreeding (Additional Figs. 2 to 8 and Fig. 1). This variance corresponded to 41 to 48% and 25 to 64% of the epistatic variance in the noninbred and inbred populations, respectively. Concerning the genetic covariances, in general, they showed lower magnitudes relative to the genetic variances and positive and negative signs, regardless of the type of epistasis, percentage of interacting genes, LD level, and degree of inbreeding (Additional Figs. 2 to 8 and Fig. 1). The sum of the covariances achieved significant values with inbreeding and 100% epistatic genes. The total value was positive under dominant epistasis and negative for the other epistasis types.

**Fig. 2:**
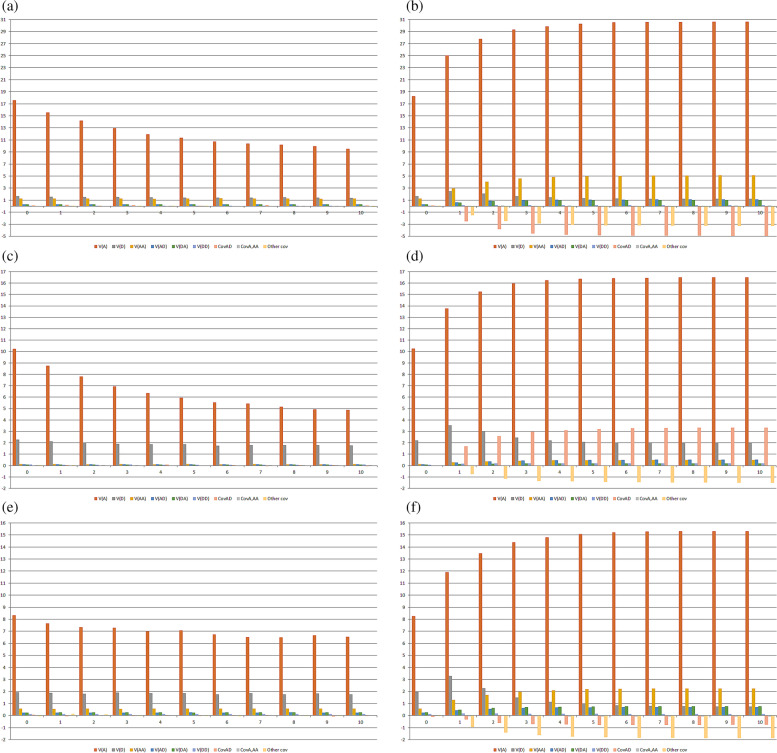
Components of the genotypic variance in the not improved (**a** and **b**) and improved (**c** and **d**) populations, both with intermediate LD level, and in the population with low LD level (**e** and **f**), along 10 generations of random crosses (**a**, **c**, and **e**) or selfing (**b**, **d**, and **f**), assuming an admixture of digenic epistasis, 30% epistatic genes, and sample size of 5000 per generation

In addition to the LD level and degree of inbreeding, the type of epistasis, percentage of interacting genes, and average allelic frequencies affect the magnitude of the epistatic variances. The epistatic variance/genotypic variance ratio was maximized under duplicate and dominant epistasis and minimized assuming recessive and complementary epistasis irrespective of the percentage of interacting genes (Additional Figs. 2 to 8 and Fig. 1). Increasing the percentage of epistatic genes from 30 to 100% increased the epistatic variance/genotypic variance ratio by a rate of 1.3 to 12.6, especially in inbred populations. Fixing the LD level at an intermediate level and assuming an admixture of epistasis types and 30% interacting genes, the epistatic variance/genotypic variance ratio was maximized in the population with an average allelic frequency for the dominant genes of 0.3, relative to the population with an average allelic frequency of 0.7, especially in the noninbred populations (Fig. 2). Thus, increasing the average allelic frequency from 0.3 to 0.7 decreased in approximately − 70% the epistatic variance/genotypic variance ratio in the noninbred populations but lead to a slight increase of the ratio in the inbred populations. The epistatic variance/genotypic variance ratios in the noninbred and inbred populations (ranges of 9 to 15% and 9 to 10%, respectively) were greater than the ratios in the population with high LD and an average allelic frequency of 0.5 (ranges of 3 to 8% for both random cross and selfing generations). But they are comparable to the ratios in the noninbred population with low LD and an average allelic frequency of 0.5 along the generations of random crosses (9 to 12%). In the inbred generations from the low LD population with an average allelic frequency of 0.5, the epistatic variance/genotypic variance ratio ranged from 10 to 22%. Decreasing the LD level from high to low, under an average allelic frequency of 0.5, maximized the epistatic variance/genotypic variance ratio in both noninbred and inbred populations. The increases ranged from 66 to 238%, especially in inbred populations. Thus, under positive dominance, the epistatic variance/genotypic variance ratio was maximized in the noninbred and inbred populations with intermediate LD and an average allelic frequency for the dominant genes of 0.3 and in the noninbred and inbred populations with low LD and an average allelic frequency of 0.5.

## Discussion

WG Hill, ME Goddard and PM Visscher [10] emphasize that knowledge about the relative magnitudes of the additive, dominance, and epistatic variances is important in evolutionary biology, medicine, and agriculture. However, the theoretical investigation about the joint significance of LD, epistasis, and inbreeding on the genetic variances for a quantitative trait is a challenge, even when fixing the number of genes, the allelic frequencies, and the degrees of dominance. One main reason is that the theory available is too complex to allow the assessment of the relative magnitudes of the genetic variances [3, 4, 10, 19]. The other main reason is the large number of combinations between levels of LD (low to high) and inbreeding (not inbred to completely inbred) with distinct percentages of epistatic genes (for example, 30 to 100%), degree of epistasis (digenic to a high order), and type of epistasis (up to seven types of digenic epistasis, complementary or duplicate trigenic or high-order epistasis, or an admixture of types).

BS Weir and CC Cockerham [18] derived very complex functions for the components of the genotypic variance assuming a two-gene model with inbreeding, LD, and epistasis and concluded that the functions are of “little use”. T Wang and ZB Zeng [19] highlight that their theoretical results serve only as a framework to understand and properly interpret estimates of the genetic effects and variance components in a QTL mapping experiment. The theoretical models investigated by WG Hill, ME Goddard and PM Visscher [10], assuming linkage equilibrium, predict high proportions of additive variance even in the presence of non-additive gene action. Assuming linkage equilibrium, the theoretical results from A Maki-Tanila and WG Hill [4] showed that the epistatic variance is of low magnitude compared with the additive variance, even when assuming high heterozygosity. They also emphasize that the majority of the epistatic variance is due to two-locus interactions. Based on theoretical models including LD, WG Hill and A Maki-Tanila [3] confirmed that most of the genotypic variance in a segregating population is additive.

Because the main conclusion from the previously described studies is that most of the genotypic variance is additive, we believe that our simulation-based study provides significant additional knowledge about the influence of LD, inbreeding and epistasis on genetic variances. Our study has a strong theoretical background on quantitative genetics for modelling LD and epistasis. We assumed low to high LD levels for genes, not inbred to completely inbred populations, 30 and 100% epistatic genes, and the seven types of digenic epistasis. Although there is evidence for higher-order epistasis, pairwise interactions contribute substantially to phenotypic variation between individuals [4, 20].

Our results agree with the main conclusions from WG Hill and A Maki-Tanila [3], A Maki-Tanila and WG Hill [4], and WG Hill, ME Goddard and PM Visscher [10], showing that LD significantly affects genetic variances and that most of the genotypic variance is additive. However, from the analyses assuming an admixture of the types of epistasis and 30% of interacting genes, the epistatic variance/genotypic variance ratio was maximized in the populations with intermediate LD and an average allelic frequency of 0.3 for the dominant genes and with low LD and an average frequency of 0.5 regardless of the generation and degree of inbreeding. The ratio was minimized in the populations with intermediate LD and an average allelic frequency for the dominant genes of 0.7 and with high LD and an average frequency of 0.5. Our results also give support to the main conclusions of J Clo, J Ronfort and D Abu Awad [2], who assumed an additive model under LD and distinct selfing rates. The differences observed for outcrossing species rely on their assumption of negative LD.

This study and the investigations of WG Hill and A Maki-Tanila [3], A Maki-Tanila and WG Hill [4], and WG Hill, ME Goddard and PM Visscher [10] show that the impact of LD, epistasis, and inbreeding on genetic variances depends on the LD level, predominant type and order of epistasis, percentage of epistatic genes, magnitude of the epistatic effects, average allelic frequency, and degree of inbreeding. The results based on simulated datasets agree with the results from QTL mapping based on field data and with the current knowledge about biological pathways and gene networks. They indicate that epistasis is important in determining quantitative traits for a range of species and traits, and that the genotypic variance is most additive [9]. Because only in specific situations can the epistatic variance constitute a high proportion of the genotypic variance, the simulated results explain why is difficult to detect epistasis, especially under small sample size [9].

In random cross populations, as human subpopulations, there are variable degree of LD [21] but inbreeding coefficients close to zero even when the subpopulation has a limited effective size or a rate of consanguineous mattings higher than that expected under random crosses [22]. This imply that in random cross populations, LD but not inbreeding can significantly affects the genotypic variance and the covariance between relatives. In noninbred populations under recurrent selection, this implies that the emphasis should continue to be selecting based on estimated/predicted breeding values even when fitting the additive-model. In self-pollinated crops, the simulated results show that the epistatic variance can constitute a significant fraction of the genotypic variance, especially under a high percentage of interacting genes. But, the main component of the epistatic variance is due to additive x additive effects. Because the covariance between parent and offspring depends mainly on additive and additive x additive effects, selecting based on estimated/predicted breeding values from fitting the additive-dominance with additive x additive epistasis model should be effective. Recognizing that epistasis can be an important effect determining quantitative traits, several recently studies on genomic selection and GWAS included epistatic effects, aiming to increase the prediction accuracy and the power of QTL detection [11–14]. In these studies, the epistatic variance ranged from 0 to 9.5% of the phenotypic variance.

## Conclusions

Additive variance is in general the most important component of genotypic variance. LD and inbreeding have a significant effect on the magnitude of the genetic variances and covariances. In general, the additive x additive variance is the most important component of the epistatic variance. The maximization of the epistatic variance/genotypic variance ratio depends on the LD level, degree of inbreeding, epistasis type, percentage of interacting genes, and average allelic frequency.

## Methods

### Additive and dominance genetic values in inbred populations

Assume initially a single biallelic gene (A/a) determining a quantitative trait, where A is the gene that increases the trait expression, and a population derived by n generations of selfing from a Hardy-Weinberg equilibrium population (generation 0). Defining ${M}_F^1$ and ${M}_F^2$ as the means of the inbred population after an allelic substitution for the genes A and a, respectively, the average effect of the allelic genes in the inbred population are ${\alpha}_A^{(n)}={M}_F^1-{M}_F= q\alpha +2 Fpqd$ and ${\alpha}_a^{(n)}={M}_F^2-{M}_F=- p\alpha +2 Fpqd$, where *M*_*F*_ = *m* + (*p* − *q*)*a* + 2*pqd* − 2*Fpqd* = *M* − 2*Fpqd* is the inbred population mean, p and q are the allelic frequencies, *α* is the average effect of an allelic substitution, *F* is the inbreeding coefficient, and M is the noninbred population mean. Thus, the additive values in the inbred population are ${A}_{AA}^{(n)}=2 q\alpha +4 Fpqd={A}_{AA}^{(0)}+4 Fpqd$, ${A}_{Aa}^{(n)}=\left(q-p\right)\alpha +4 Fpqd={A}_{Aa}^{(0)}+4 Fpqd$, and ${A}_{aa}^{(n)}=-2 p\alpha +4 Fpqd={A}_{aa}^{(0)}+4 Fpqd$, where *A*^(0)^ is the additive value in the noninbred population. Note that *E*(*A*^(*n*)^) = 4*Fpqd*. Expressing the genotypic values in the inbred population as a function of *M*_*F*_, we have:

$${G}_{AA}={M}_F+{A}_{AA}^{(0)}+\left(-2{q}^2d+2 Fpqd\right)={M}_F+{A}_{AA}^{(0)}+\left({D}_{AA}^{(0)}+2 Fpqd\right)={M}_F+{A}_{AA}^{(0)}+{D}_{AA}^{(n)}$$

$${G}_{Aa}={M}_F+{A}_{Aa}^{(0)}+\left(2 pqd+2 Fpqd\right)={M}_F+{A}_{Aa}^{(0)}+\left({D}_{Aa}^{(0)}+2 Fpqd\right)={M}_F+{A}_{Aa}^{(0)}+{D}_{Aa}^{(n)}$$

$${G}_{aa}={M}_F+{A}_{aa}^{(0)}+\left(-2{p}^2d+2 Fpqd\right)={M}_F+{A}_{aa}^{(0)}+\left({D}_{aa}^{(0)}+2 Fpqd\right)={M}_F+{A}_{aa}^{(0)}+{D}_{aa}^{(n)}$$

Note that in the inbred population, *E*(*A*^(0)^) = *E*(*D*^(*n*)^) = 0 but *E*(*D*^(0)^) =  − 2*Fpqd*. Note also that the additive value in the noninbred population is the additive value in the inbred population expressed as deviation from its mean (*A*^(0)^ = *A*^(*n*)^ − 4*Fpqd*) and the dominance value in the inbred population is the dominance value in the noninbred population expressed as deviation from its mean (*D*^(*n*)^ = *D*^(0)^ + 2*Fpqd*). This implies that, in the inbred population, *E*(*G*) = *M*_*F*_.

### Genetic variances in inbred populations in LD

Assume now two linked biallelic genes (A/a and B/b) determining a quantitative trait and a noninbred population in LD (generation 0). Assume dominance but initially no epistasis. After n generations of selfing, the genotypic variance for the two genes in the inbred population is (see the genotype probabilities in the Additional File Appendix) ${\sigma}_G^{2(n)}={\sigma}_A^{2(n)}+{\sigma}_D^{2(n)}+2{\sigma}_{A,D}^{(n)}$, where:

$${\sigma}_A^{2(n)}=\left(1+F\right)\left(2{p}_a{q}_a{\alpha}_a^2+2{p}_b{q}_b{\alpha}_b^2\right)+2\left[2+{c}_1\left(1-2{r}_{ab}\right)\right]{\Delta }_{ab}^{\left(-1\right)}{\alpha}_a{\alpha}_b=\left(1+F\right){\sigma}_A^{2(0)}+2\left[{c}_1\left(1-2{r}_{ab}\right)-2F\right]{\Delta }_{ab}^{\left(-1\right)}{\alpha}_a{\alpha}_b$$

is the additive variance,

$${\sigma}_D^{2(n)}=\left(1-{F}^2\right)\left(4{p}_a^2{q}_a^2{d}_a^2+4{p}_b^2{q}_b^2{d}_b^2\right)+F\left[4{p}_a{q}_a{\left({p}_a-{q}_a\right)}^2{d}_a^2+4{p}_b{q}_b{\left({p}_b-{q}_b\right)}^2{d}_b^2\right]+8\left\{\left(1-F\right)\left({c}^n-1+F\right){p}_a{q}_a{p}_b{q}_b+\left({p}_a-{q}_a\right)\left({p}_b-{q}_b\right)\left[\left(1-F\right){c}^n-\left(1-2F\right)+{c}_1\left(1-2{r}_{ab}\right)/2\right]{\Delta }_{ab}^{\left(-1\right)}/2+\left(1-F\right){c}^n{\Delta _{ab}^{\left(-1\right)}}^2\right\}{d}_a{d}_b=\left(1-{F}^2\right){\sigma}_D^{2(0)}+F{D}_2+8\left\{\left(1-F\right)\left({c}^n-1+F\right){p}_a{q}_a{p}_b{q}_b+\left({p}_a-{q}_a\right)\left({p}_b-{q}_b\right)\left[\left(1-F\right){c}^n-\left(1-2F\right)+{c}_1\left(1-2{r}_{ab}\right)/2\right]{\Delta }_{ab}^{\left(-1\right)}/2+\left[\left(1-F\right){c}^n-\left(1-{F}^2\right)\right]{\Delta _{ab}^{\left(-1\right)}}^2\right\}{d}_a{d}_b$$

is the dominance variance, and

$${\sigma}_{A,D}^{(n)}=2F\left[2{p}_a{q}_a\left({p}_a-{q}_a\right){\alpha}_a{d}_a+2{p}_b{q}_b\left({p}_b-{q}_b\right){\alpha}_b{d}_b\right]+\left[2F+{c}_1\left(1-2{r}_{ab}\right)\right]{\Delta }_{ab}^{\left(-1\right)}\left[\left({p}_b-{q}_b\right){\alpha}_a{d}_b+\left({p}_a-{q}_a\right){\alpha}_b{d}_a\right]=2F{D}_1+\left[2F+{c}_1\left(1-2{r}_{ab}\right)\right]{\Delta }_{ab}^{\left(-1\right)}\left[\left({p}_b-{q}_b\right){\alpha}_a{d}_b+\left({p}_a-{q}_a\right){\alpha}_b{d}_a\right]$$

is the covariance between additive and dominance values,

where ${\Delta }_{ab}^{\left(-1\right)}={P}_{AB}^{\left(-1\right)}.{P}_{ab}^{\left(-1\right)}-{P}_{Ab}^{\left(-1\right)}.{P}_{aB}^{\left(-1\right)}$ is the measure of LD in the gametic pool of generation −1 [23], where *P*^(−1)^ is a haplotype probability, *r*_*ab*_ is the recombination frequency, *c*_1_ = 2{1 − [(1 − 2*r*_*ab*_)/2]^*n*^}/(1 + 2*r*_*ab*_), *c* = 1 − 2*r*_*ab*_(1 − *r*_*ab*_), ${\sigma}_A^{2(0)}=2{p}_a{q}_a{\alpha}_a^2+2{p}_b{q}_b{\alpha}_b^2+4{\Delta }_{ab}^{\left(-1\right)}{\alpha}_a{\alpha}_b$ and ${\sigma}_D^{2(0)}=4{p}_a^2{q}_a^2{d}_a^2+4{p}_b^2{q}_b^2{d}_b^2+8{\Delta }_{ab}^{\left(-1\right)}{d}_a{d}_b$ are the additive and dominance variances in the noninbred population in LD [24], and *D*_1_ (covariance of a and d) and *D*_2_ (variance of d) are the components of the covariance of relatives from self-fertilization, assuming linkage equilibrium [8]. The other terms are the covariances between the average effects of an allelic substitution, between dominance deviations, and between the average effect of an allelic substitution and dominance deviation, for genes in LD. Because we assumed biallelic genes, $\overset{\check{} }{H}={\sigma}_D^2.$ Thus, $\left(1-{F}^2\right){\sigma}_D^{2(0)}=\left(1-F\right){\sigma}_D^{2(0)}+F\left(1-F\right)\overset{\check{} }{H}$. Note that the genotypic variance derived here is a general formulation for the Cockerham’s genotypic variance c_ggg_ [8], assuming LD. If p = q, ${\sigma}_{A,D}^{(n)}=0$.

Assuming LD but no inbreeding, the genotypic variance after n generations of random cross in the noninbred population in LD is ${\sigma}_G^{2(n)}={\sigma}_A^{2(n)}+{\sigma}_D^{2(n)}$, because ${\sigma}_{A,D}^{(n)}=0$, where:

$${\sigma}_A^{2(n)}=2{p}_a{q}_a{\alpha}_a^2+2{p}_b{q}_b{\alpha}_b^2+4{\left(1-{r}_{ab}\right)}^n{\Delta }_{ab}^{\left(-1\right)}{\alpha}_a{\alpha}_b$$

$${\sigma}_D^{2(n)}=4{p}_a^2{q}_a^2{d}_a^2+4{p}_b^2{q}_b^2{d}_b^2+8{\left[{\left(1-{r}_{ab}\right)}^n{\Delta }_{ab}^{\left(-1\right)}\right]}^2{d}_a{d}_b$$

Thus, the genotypic variance can increase or decrease after n generations of random cross in a noninbred population, depending on the sign of the LD measure. The LD value is positive for genes in coupling phase and negative for genes in repulsion phase.

### Epistasis in noninbred and inbred populations in LD

The quantitative genetics theory for modelling epistasis in a population in LD is a generalization of the theory proposed by O Kempthorne [16], who assumed a noninbred population in linkage equilibrium and any number of alleles. We assumed biallelism. It should be emphasized that the Kempthorne’s theory allows a generalization from two to three or more interacting genes. But fitting three or more interacting genes in a population in LD is a challenge because the genotype probabilities for three or more genes in LD are too complex to derive. Furthermore, only complementary and duplicate epistasis can be easily defined for three or more epistatic genes.

Assume now that the two previous defined genes are epistatic. The genotypic value is [16]:

$${G}_{ij k l}=M+{\alpha}_i^1+{\alpha}_j^1+{\alpha}_k^2+{\alpha}_l^2+{\delta}_{ij}^1+{\delta}_{kl}^2+{\left({\alpha}^1{\alpha}^2\right)}_{ik}+{\left({\alpha}^1{\alpha}^2\right)}_{jk}+{\left({\alpha}^1{\alpha}^2\right)}_{il}+{\left({\alpha}^1{\alpha}^2\right)}_{jl}+{\left({\alpha}^1{\delta}^2\right)}_{ik l}+{\left({\alpha}^1{\delta}^2\right)}_{jk l}+{\left({\delta}^1{\alpha}^2\right)}_{ij k}+{\left({\delta}^1{\alpha}^2\right)}_{ij l}+{\left({\delta}^1{\delta}^2\right)}_{ij k l}=M+A+D+ AA+ AD+ DA+ DD$$

where AA, AD, DA, and DD are the additive x additive, additive x dominance, dominance x additive, and dominance x dominance epistatic genetic values.

The parametric values of the 36 parameters for the nine genotypic values are obtained by solving the equations *β* = (*X* ′ *VX*)^−1^*X* ′ *Vy*, under the restrictions defined by O Kempthorne [16], where *X* is the incidence matrix, $V= diagonal\left\{{f}_{ij}^{(n)}\right\}$ is the diagonal matrix of the genotype probabilities, and *y* is the vector of the genotypic values (*G*_*ij*_) (i, j = 0, 1, and 2).

O Kempthorne [16] provided explicit functions for all effects because he assumed linkage equilibrium. Assuming LD makes very difficult to derive such functions but the following results hold:

1. the expectation of the breeding value is zero regardless of the degree of inbreeding in the population.
2. the expectation of the dominance value is *E*(*D*)^(*n*)^ = *p*_*a*_*q*_*a*_*F*(*δ*_*AA*_ − 2*δ*_*Aa*_ + *δ*_*aa*_) + *p*_*b*_*q*_*b*_*F*(*δ*_*BB*_ − 2*δ*_*Bb*_ + *δ*_*bb*_); then, defining the dominance value in an inbred population as the dominance value expressed as deviation from its mean (*D*^(*n*)^ = *D* − *E*(*D*)^(*n*)^), *E*(*D*^(*n*)^) = 0.
3. the expectation of the additive x additive value is zero only if there is no LD.
4. the expectation of the additive x dominance value is zero only if F = 0 or p = q for all genes.
5. the expectation of the dominance x additive value is zero only if F = 0 or p = q for all genes.
6. the expectation of the dominance x dominance value is zero only if F = 0 and there is no LD.

Thus, defining the additive x additive, additive x dominance, dominance x additive, and dominance x dominance epistatic values as the values expressed as deviation from its mean, *AA*^(*n*)^ = *AA* − *E*(*AA*)^(*n*)^, *AD*^(*n*)^ = *AD* − *E*(*AD*)^(*n*)^, *DA*^(*n*)^ = *DA* − *E*(*DA*)^(*n*)^, and *DD*^(*n*)^ = *DD* − *E*(*DD*)^(*n*)^, the genotypic value in an inbred population can be expressed as

$$G=M+E{(D)}^{(n)}+E{(AA)}^{(n)}+E{(AD)}^{(n)}+E{(DA)}^{(n)}+E{(DD)}^{(n)}+A+{D}^{(n)}+{AA}^{(n)}+{AD}^{(n)}+{DA}^{(n)}+{DD}^{(n)}={M}_F+A+{D}^{(n)}+{AA}^{(n)}+{AD}^{(n)}+{DA}^{(n)}+{DD}^{(n)}$$

This implies that *E*(*G*) = *M*_*F*_. If F = 0 then

$$G=M+E(AA)+E(DD)+A+D+\left[ AA-E(AA)\right]+ AD+ DA+\left[ DD-E(DD)\right]={M}^{\ast }+A+D+{AA}^{\ast }+ AD+ DA+{DD}^{\ast }$$

where,

$$E(AA)=2\triangle_{ab}^{\left(-1\right)}(\alpha_A\alpha_B-\alpha_A\alpha_b-\alpha_a\alpha_B+\alpha_a\alpha_b)$$

and

$$E(DD)=\left[\triangle_{ab}^{\left(-1\right)}\right]^2\;(\delta_{AA}\delta_{BB}-2\delta_{AA}\delta_{Bb}+\delta_{AA}\delta_{bb}-2\delta_{Aa}\delta_{BB}+4\delta_{Aa}\delta_{Bb}-2\delta_{Aa}\delta_{bb}+\delta_{aa}\delta_{BB}-2\delta_{aa}\delta_{Bb}+\delta_{aa}\delta_{bb})$$

This implies that $E(G)=M^*$. If F = 0 and there is no LD,

$$G=M+A+D+ AA+ AD+ DA+ DD$$

where the linear components are those defined by O Kempthorne [16]. This implies that *E*(*G*) = *M*.

In noninbred populations in LD, only the additive and dominance values are not correlated. The genotypic variance in these populations is, in simplified form,

$${\sigma}_G^{2(0)}={\sigma}_A^{2(0)}+{\sigma}_D^{2(0)}+{\sigma}_{AA}^{2(0)}+2{\sigma}_{A, AA}^{(0)}+2{\sigma}_{D, AA}^{(0)}+\dots$$

where

$${\sigma}_{AA}^{2(0)}={f}_{22}^{(0)}{\left[\left(4{\alpha}_A{\alpha}_B\right)\right]}^2+\dots +{f}_{00}^{(0)}{\left[\left(4{\alpha}_a{\alpha}_b\right)\right]}^2-{\left[E{(AA)}^{(0)}\right]}^2$$

$${\sigma}_{A, AA}^{(0)}=2{\Delta }_{ab}^{\left(-1\right)}\left[{\alpha}^A\left({\alpha}_A{\alpha}_B-{\alpha}_A{\alpha}_b+{\alpha}_a{\alpha}_B-{\alpha}_a{\alpha}_b\right)+{\alpha}^B\left({\alpha}_A{\alpha}_B-{\alpha}_a{\alpha}_B+{\alpha}_A{\alpha}_b-{\alpha}_a{\alpha}_b\right)\right]$$

$${\sigma}_{D, AA}^{(0)}=-4{\Delta }_{ab}^{\left(-1\right)}\left[{p}_a{q}_a{d}_a\left({\alpha}_A{\alpha}_B-{\alpha}_A{\alpha}_b-{\alpha}_a{\alpha}_B+{\alpha}_a{\alpha}_b\right)+{p}_b{q}_b{d}_b\left({\alpha}_A{\alpha}_B-{\alpha}_a{\alpha}_B-{\alpha}_A{\alpha}_b+{\alpha}_a{\alpha}_b\right)\right]$$

where, to avoid confusion, *α*^*A*^ and *α*^*B*^ are the average effects of an allelic substitution.

The assumption of LD makes very difficult to derive the components of the genotypic variance (additive, dominance, and epistatic variances and the covariances between these effects), even assuming noninbred populations, biallelic genes, and only digenic epistasis. In respect to the types of digenic epistasis, the following can be defined [25, 26]:

1. Complementary (*G*_22_ = *G*_21_ = *G*_12_ = *G*_11_ and *G*_20_ = *G*_10_ = *G*_02_ = *G*_01_ = *G*_00_; proportion of 9:7 in a F_2_, assuming independent assortment).
2. Duplicate (*G*_22_ = *G*_21_ = *G*_20_ = *G*_12_ = *G*_11_ = *G*_10_ = *G*_02_ = *G*_01_; proportion of 15:1 in a F_2_).
3. Dominant (*G*_22_ = *G*_21_ = *G*_20_ = *G*_12_ = *G*_11_ = *G*_10_ and *G*_02_ = *G*_01_; proportion of 12:3:1 in a F_2_).
4. Recessive (*G*_22_ = *G*_21_ = *G*_12_ = *G*_11_, *G*_02_ = *G*_01_, and *G*_20_ = *G*_10_ = *G*_00_; proportion of 9:3:4 in a F_2_)
5. Dominant and recessive (*G*_22_ = *G*_21_ = *G*_12_ = *G*_11_ = *G*_20_ = *G*_10_ = *G*_00_ and *G*_02_ = *G*_01_; proportion of 13:3 in a F_2_).
6. Duplicate genes with cumulative effects (*G*_22_ = *G*_21_ = *G*_12_ = *G*_11_ and *G*_20_ = *G*_10_ = *G*_02_ = *G*_01_; proportion of 9:6:1 in a F_2_).
7. Nonepistatic genic interaction (*G*_22_ = *G*_21_ = *G*_12_ = *G*_11_, *G*_20_ = *G*_10_, and *G*_02_ = *G*_01_; proportion of 9:3:3:1 in a F_2_).

### Simulated datasets

Because the magnitude of the components of genotypic variance generally cannot be inferred from previous functions, all means and genetic variances and covariances were computed from simulated datasets provided by *REALbreeding* software (available upon request). This program uses the quantitative genetics theory that was described in the previous sections and in JMS Viana [24]. *REALbreeding* has been used to provide simulated data in investigations in the areas of genomic selection [27], GWAS [28], QTL mapping [29], linkage disequilibrium [30], population structure [31], and heterotic grouping/genetic diversity [32].

The program simulates individual genotypes for genes and molecular markers and phenotypes in three steps using user inputs. The first step (genome simulation) is the specification of the number of chromosomes, molecular markers, and genes as well as marker type and density. The second step (population simulation) is the specification of the population(s) and sample size or progeny number and size. A population is characterized by the average frequency for the genes (biallelic) and markers (first allele). The final step (trait simulation) is the specification of the individual phenotypes. In this stage, the user informs the minimum and maximum genotypic values for homozygotes (to compute the a deviations), the minimum and maximum phenotypic values (to avoid outliers), the direction and degree of dominance (to compute the dominance deviations/d), and the broad sense heritability. The current version allows the inclusion of digenic epistasis, gene x environment interaction, and multiple traits (up to 10), including pleiotropy. The population mean (M) and additive (A), dominance (D), and epistatic (AA, AD, DA, and DD) genetic values or general and specific combining ability effects (GCA and SCA) and epistatic values (I), or genotypic values (G), depending on the population, are calculated from the parametric gene effects and frequencies and the parametric LD values. The phenotypic values (*P*) are computed assuming error effects (*E*) sampled from a normal distribution (*P* = *M* + *A* + *D* + *AA* + *AD* + *DA* + *DD* + *E* = *G* + *E* or *P* = *M* + *GCA*1 + *GCA*2 + *SCA* + *I* + *E* = *G* + *E*). The population in LD is generated by crossing two populations in linkage equilibrium followed by a generation of random crosses. This generation of random crosses aims to generate a population in Hardy-Weinberg equilibrium. Thus, generation 0 (the founder population) is a population in Hardy-Weinberg equilibrium in LD for linked genes and molecular markers, and the individuals are not related. The parametric LD in this population is ${\Delta }_{ab}^{\left(-1\right)}=\left[\left(1-2{r}_{ab}\right)/4\right]\left({p}_{a1}-{p}_{a2}\right)\left({p}_{b1}-{p}_{b2}\right)$, where the indices 1 and 2 indicate the parental populations.

The quantitative genetics theory for epistasis does not solve the challenge of studying genetic variability and covariance between relatives in populations, using simulated datasets, even assuming simplified scenarios such as linkage equilibrium and no inbreeding. Because the genotypic values for any two interacting genes are not known, there are infinite genotypic values that satisfy the specifications of each type of digenic epistasis. For example, fixing the gene frequencies (the population) and the parameters m, a, d, and d/a (degree of dominance) for each gene (the trait), the solutions *G*_22_ = *G*_21_ = *G*_12_ = *G*_11_ = 5.25 and *G*_20_ = *G*_10_ = *G*_02_ = *G*_01_ = *G*_00_ = 5.71 or *G*_22_ = *G*_21_ = *G*_12_ = *G*_11_ = 6.75 and *G*_20_ = *G*_10_ = *G*_02_ = *G*_01_ = *G*_00_ = 2.71 define complementary epistasis, but the genotypic values are not the same.

The solution implemented in the software allows the user to control the magnitude of the epistatic variance (V(I)) relative to the magnitudes of the additive and dominance variances (V(A) and V(D)). As an input for the user, the software requires the ratio V(I)/(V(A) + V(D)) for each pair of interacting genes (a single value; for example, 1.0). Then, for each pair of epistatic genes the software samples a random value for the epistatic value *I*_22_ (the epistatic value for the genotype AABB), assuming *I*_22_~*N*(0, *V*(*I*)). Then, the other epistatic effects and genotypic values are computed.

We simulated grain yield (g/plant) assuming 400 genes in 10 chromosomes of 200 and 50 cM (40 genes/chromosome). The average density was approximately one gene/5 cM and one gene/cM, respectively. Assuming a density of one gene/cM, we simulated a population with an average frequency of 0.5 and high LD. Under the density of one gene/5 cM, we generated four populations, one with a high LD level and one with a low LD level, both with an average frequency of 0.5, and two populations with an intermediate LD level and an average frequency for the favorable genes of 0.3 (not improved) and 0.7 (improved). We defined positive dominance (average degree of dominance of 0.6), maximum and minimum genotypic values for homozygotes of 160 and 30 g.plt^− 1^, and maximum and minimum phenotypic values of 180 and 10 g.plt^− 1^. The broad sense heritability was 20%. For each population, we assumed additive-dominance with digenic epistasis model defining 100 and 30% of interacting genes. Concerning the ratio V(I)/(V(A) + V(D)), the analyses assuming ratios 1, 10, and 100 evidenced that increasing the ratio from 1 to 10 and 100 increased the epistatic variances but also increased the additive and dominance variances. Then, because the main conclusions for the greater ratios were essentially the same provided by ratio 1, we will present only the results for ratio 1. With epistasis, we assumed a single type or an admixture of the seven types. We ranged the degree of inbreeding from 0.0 to 1.0, assuming 10 generations of selfing. We also assumed 10 generations of random crosses. The population size was 5000 per generation.

The characterization of the LD in the populations was based on the parametric Δ, r^2^, and D′ values for the 40 genes in chromosome 1, which were provided by *REALbreeding* (it should be similar for the other chromosomes). The heatmaps were processed using the R package pheatmap. Assuming no epistasis, the software provides parametric additive and dominance genetic values and parametric genetic variances and covariances. Assuming epistasis, the software provides parametric additive, dominance, and epistatic genetic values. Thus, under epistasis, the genetic variances and covariances were computed from the parametric genetic values, using a sample size of 5000 individuals per generation.

## Supplementary Information

**Additional file 1.** Figures and Appendix.

## Data Availability

The dataset is available at 10.6084/m9.figshare.13607306.v2.

## Abbreviations

LD: Linkage disequilibrium
A: Additive value
D: Dominance value
AA: Additive x additive value
AD: Additive x dominance value
AD: Dominance x additive value
DD: Dominance x dominance value
G: Genotypic value
I: Epistatic value
