# Supplementary material for: Significance of linkage disequilibrium and epistasis on genetic variances in noninbred and inbred populations
Source: BMC Genomics. 2022 Apr 9;23:286. doi: 10.1186/s12864-022-08335-9 (PMC8994904; doi:10.1186/s12864-022-08335-9)
Supplement: Supplementary file 1 — Additional file 1. Figures and Appendix. [file 12864_2022_8335_MOESM1_ESM.pdf]

Significance of linkage disequilibrium and epistasis on the genetic variances in non-inbred and inbred populations

José Marcelo Soriano Viana and Antonio Augusto Franco Garcia

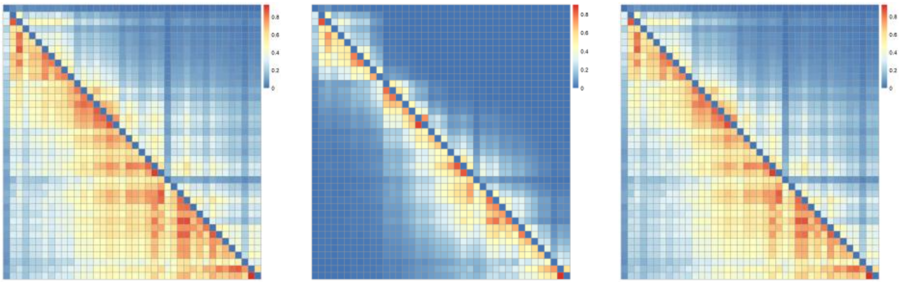

(a) (b) (c)

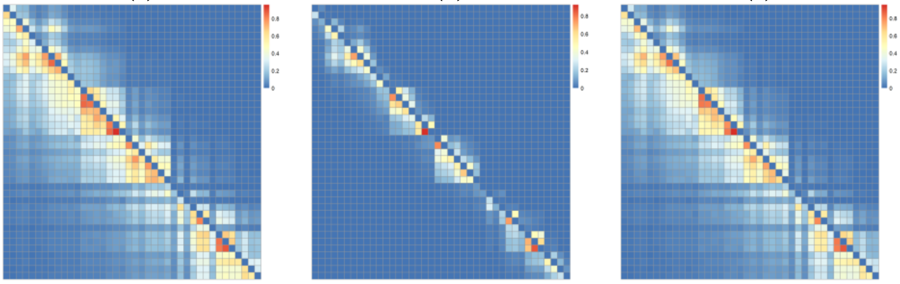

(d) (e) (f)

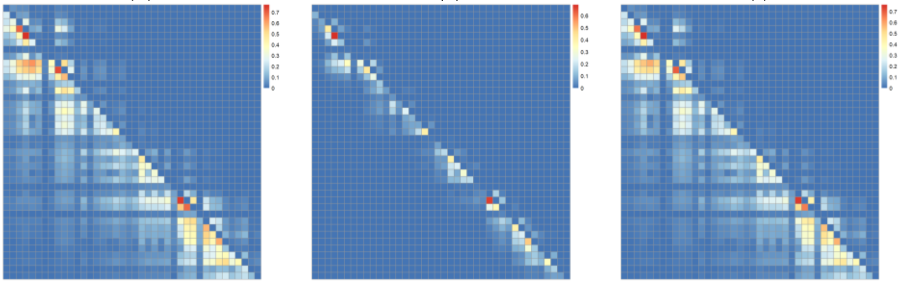

(g) (h) (i)

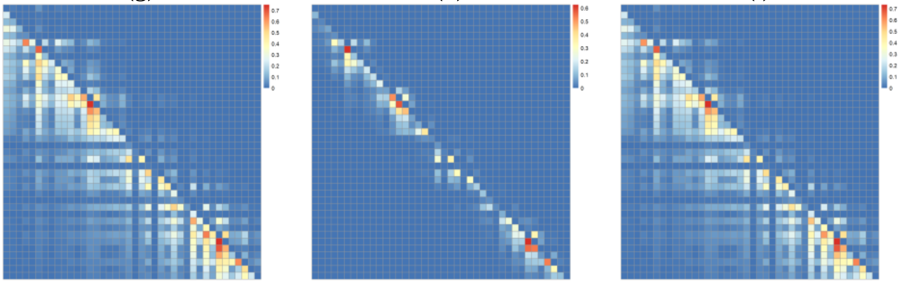

(j) (k) (l)

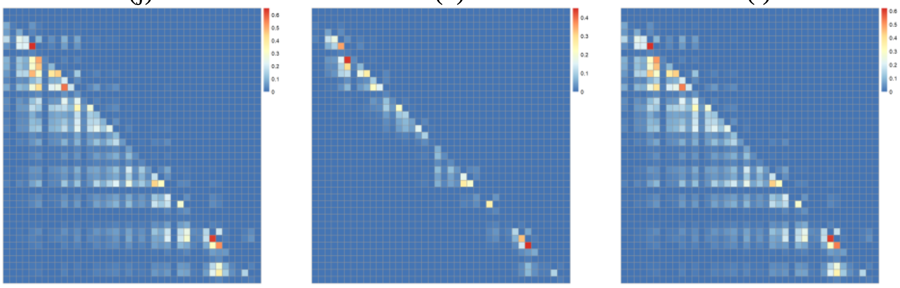

(m) (n) (o)

Additional Figure 1. Parametric r^2 (above the diagonal) and |D'| (below the diagonal) values for 40 genes in chromosome 1, in the populations with density of one gene/cM and high LD (a, b, c), and with density of one gene/5 cM and high (d, e, and f), intermediate (g, h, i, j, k, and l), and low (m, n, and o) LD, in generations 0 (a, d, g, j, and m) and 10, assuming random crosses (b, e, h, k, and n) or selfing (c, f, i, l, and o).

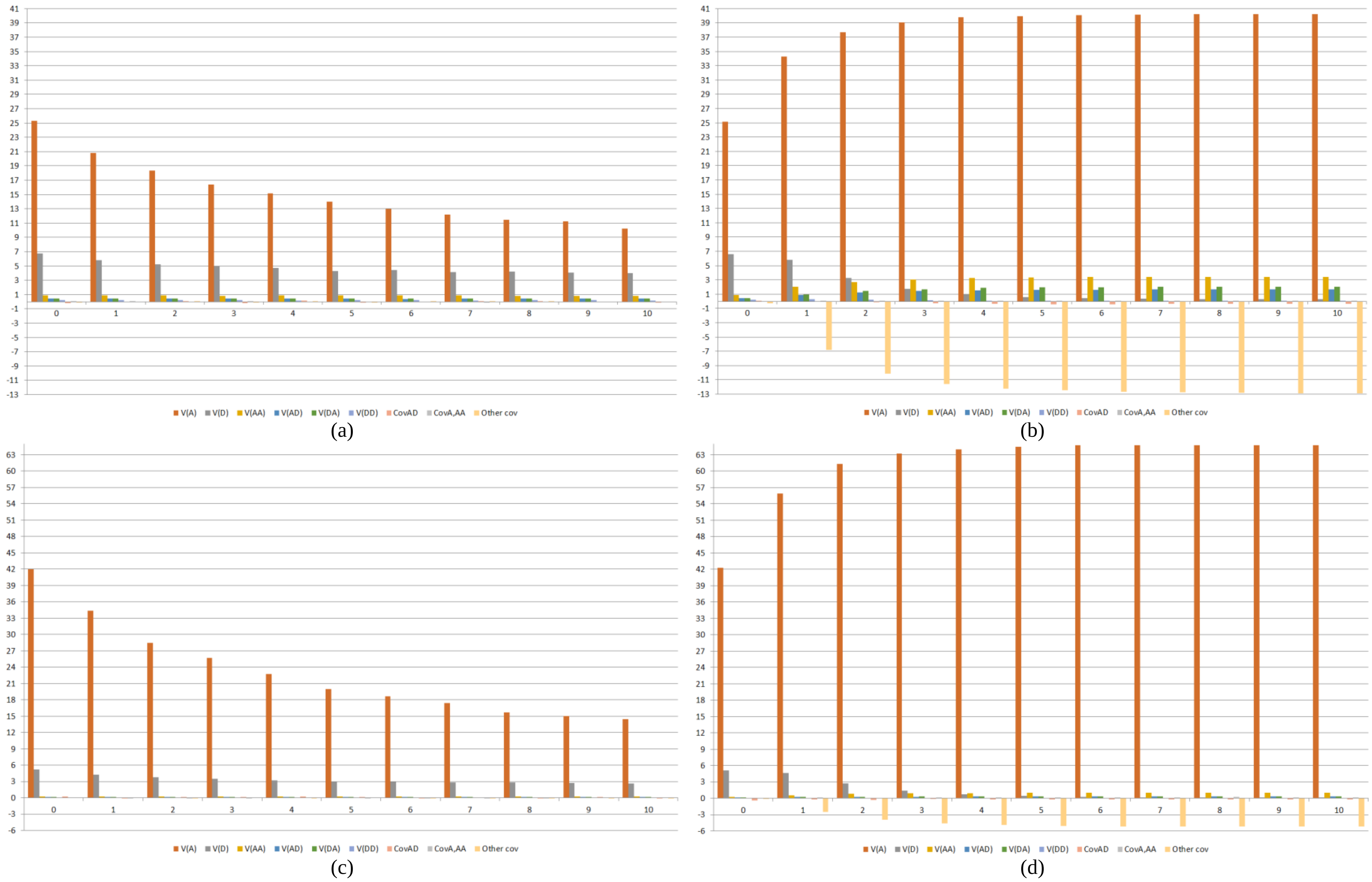

Additional Figure 2. Components of the genotypic variance in a population with high LD level, along 10 generations of random crosses (a and c) or selfing (b and d), assuming complementary epistasis, 100 (a and b) and 30% (c and d) of epistatic genes, and sample size of 5,000 per generation.

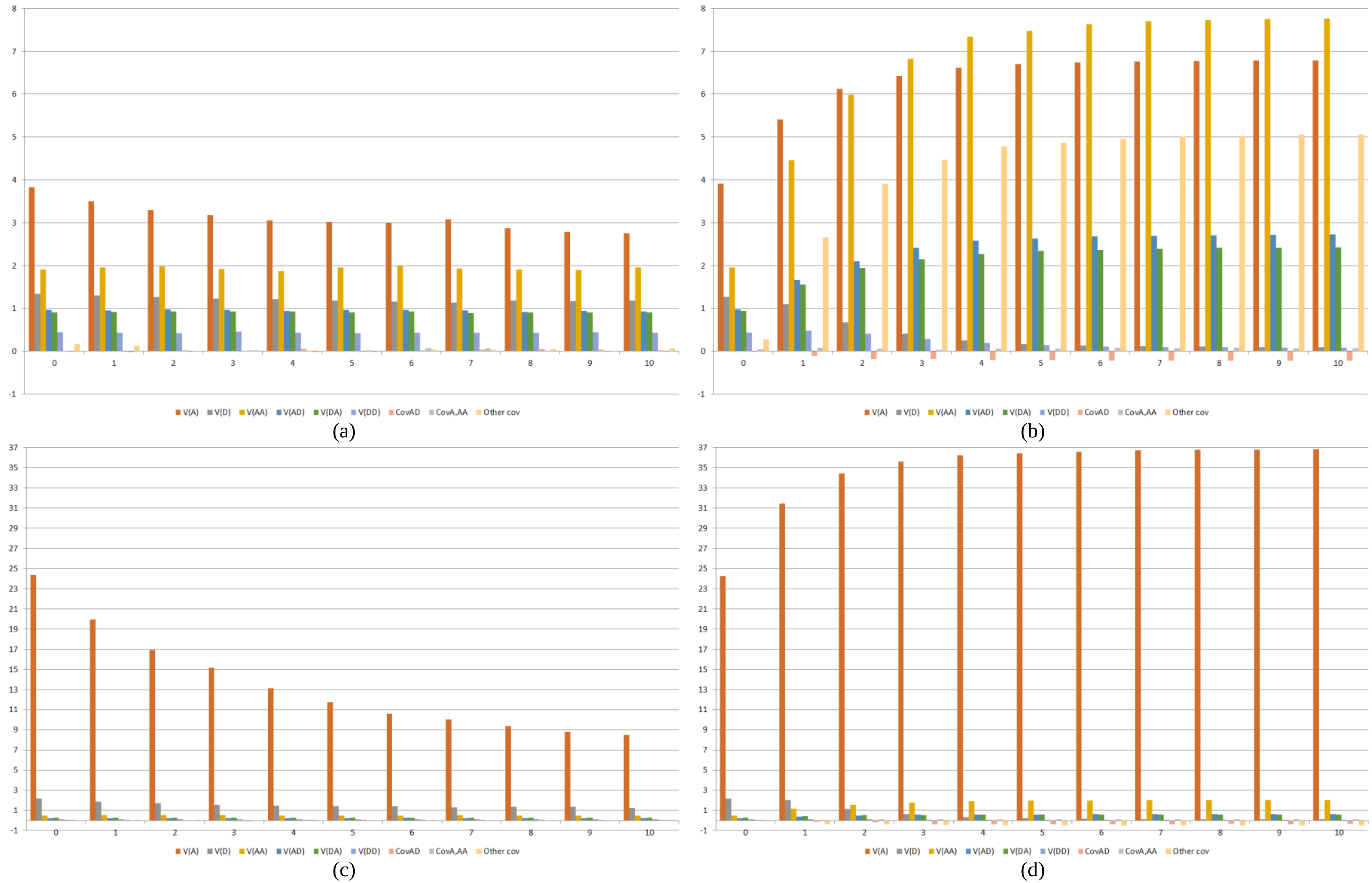

Additional Figure 3. Components of the genotypic variance in a population with high LD level, along 10 generations of random crosses (a and c) or selfing (b and d), assuming duplicate epistasis, 100 (a and b) and 30% (c and d) of epistatic genes, and sample size of 5,000 per generation.

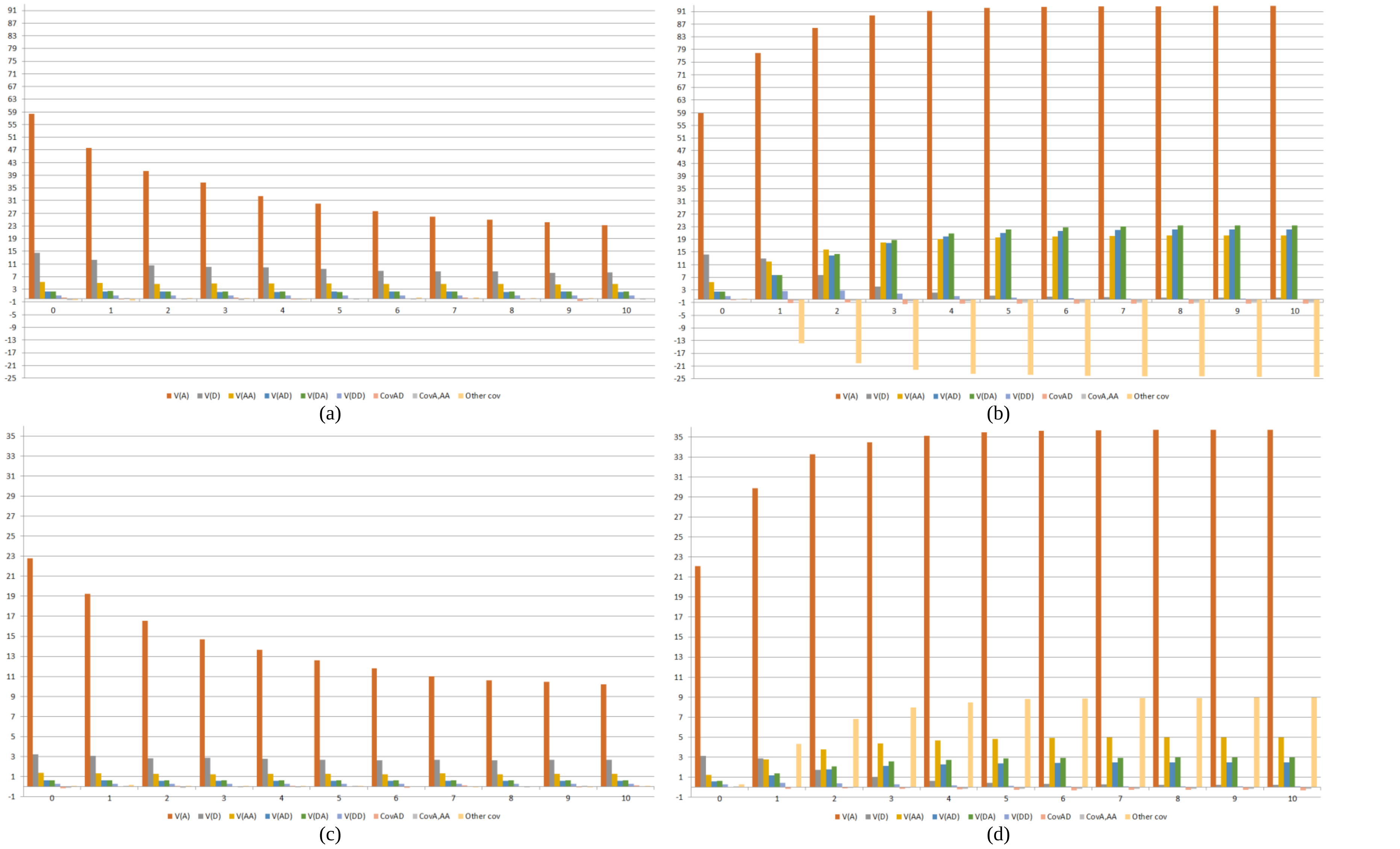

Additional Figure 4. Components of the genotypic variance in a population with high LD level, along 10 generations of random crosses (a and c) or selfing (b and d), assuming dominant epistasis, 100 (a and b) and 30% (c and d) of epistatic genes, and sample size of 5,000 per generation.

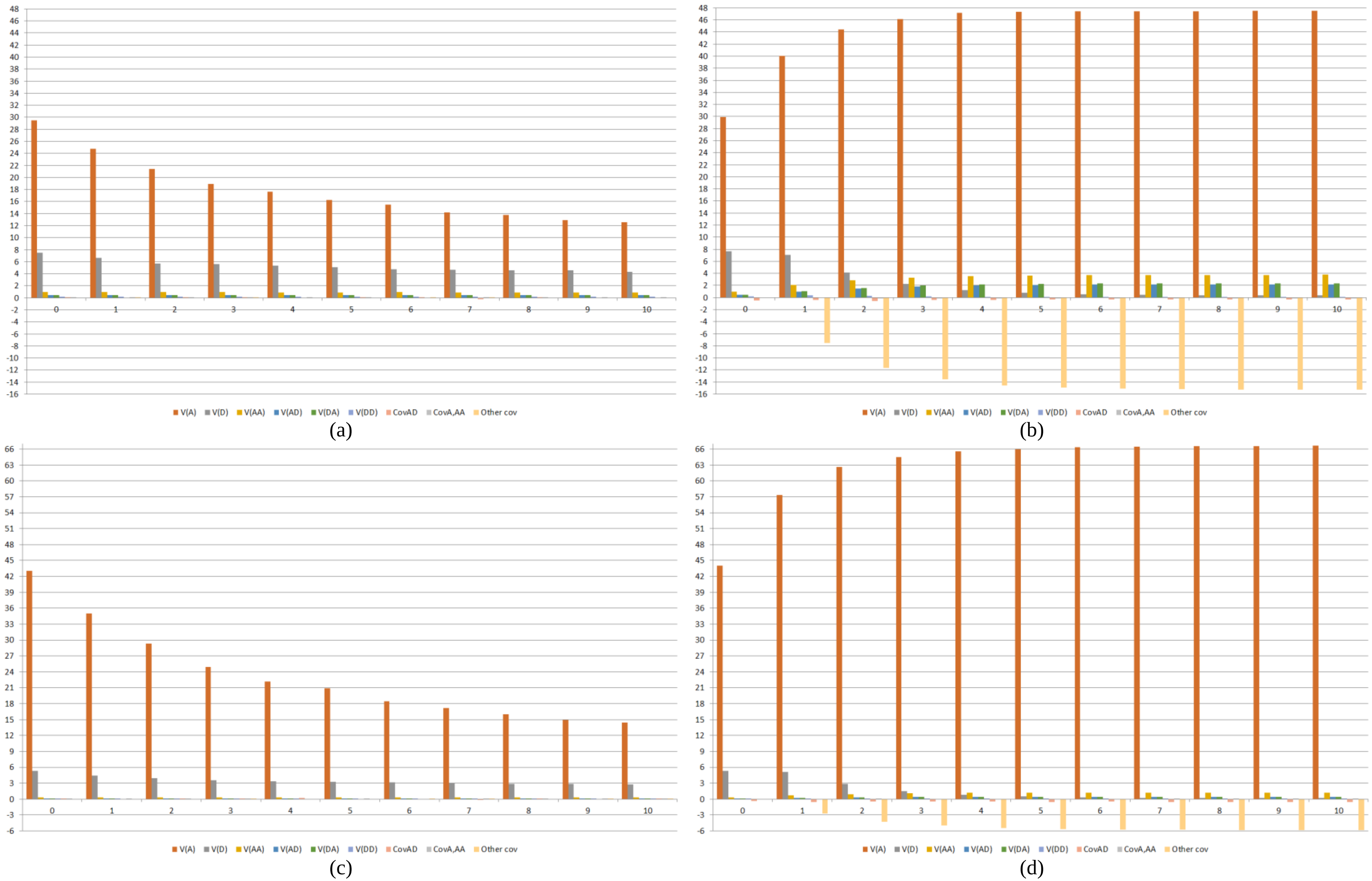

Additional Figure 5. Components of the genotypic variance in a population with high LD level, along 10 generations of random crosses (a and c) or selfing (b and d), assuming recessive epistasis, 100 (a and b) and 30% (c and d) of epistatic genes, and sample size of 5,000 per generation.

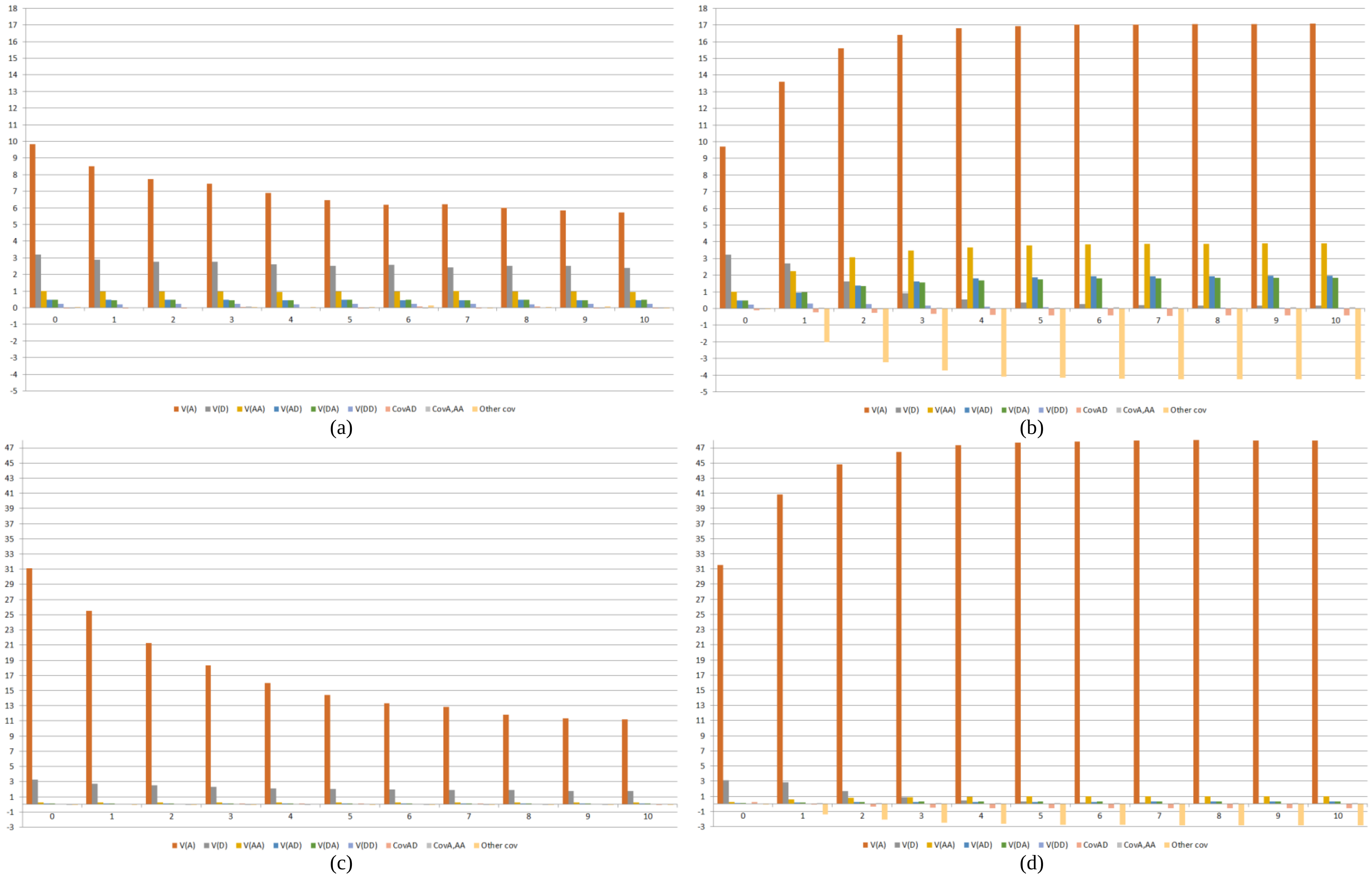

Additional Figure 6. Components of the genotypic variance in a population with high LD level, along 10 generations of random crosses (a and c) or selfing (b and d), assuming dominant and recessive epistasis, 100 (a and b) and 30% (c and d) of epistatic genes, and sample size of 5,000 per generation.

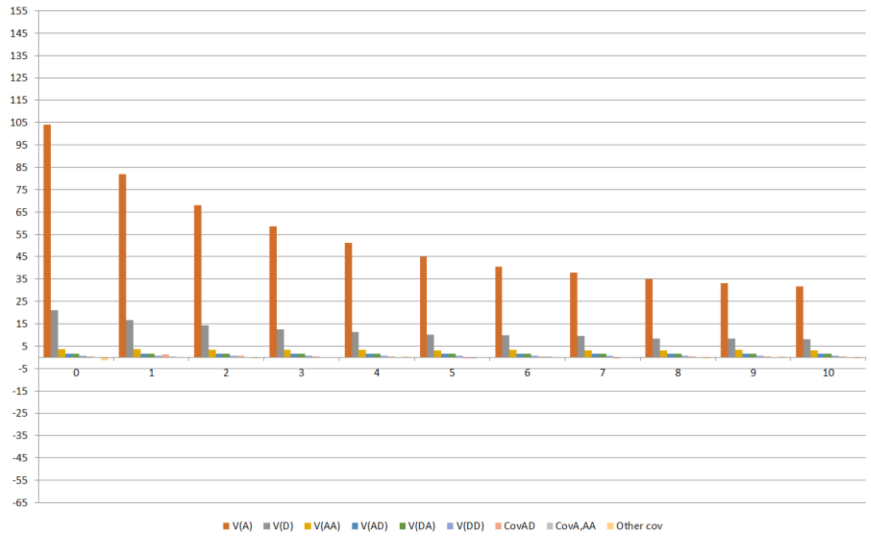

(a)

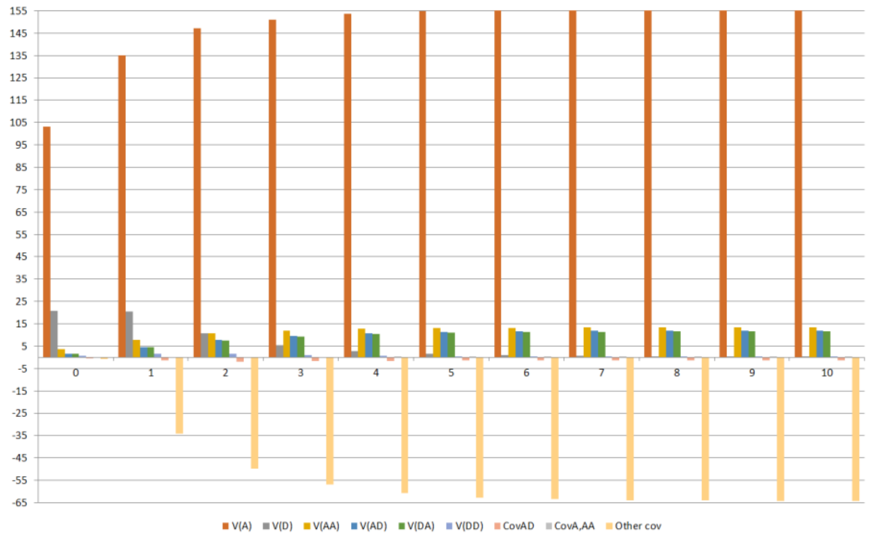

(b)

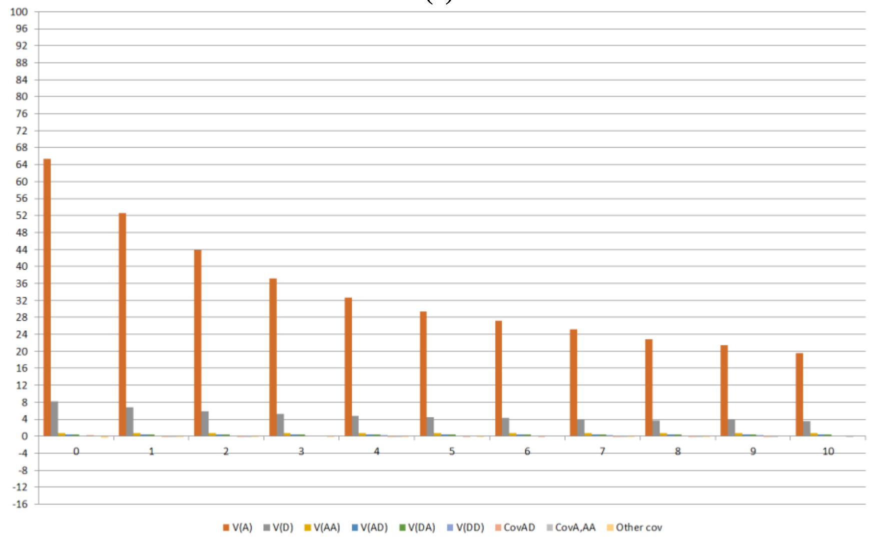

(c)

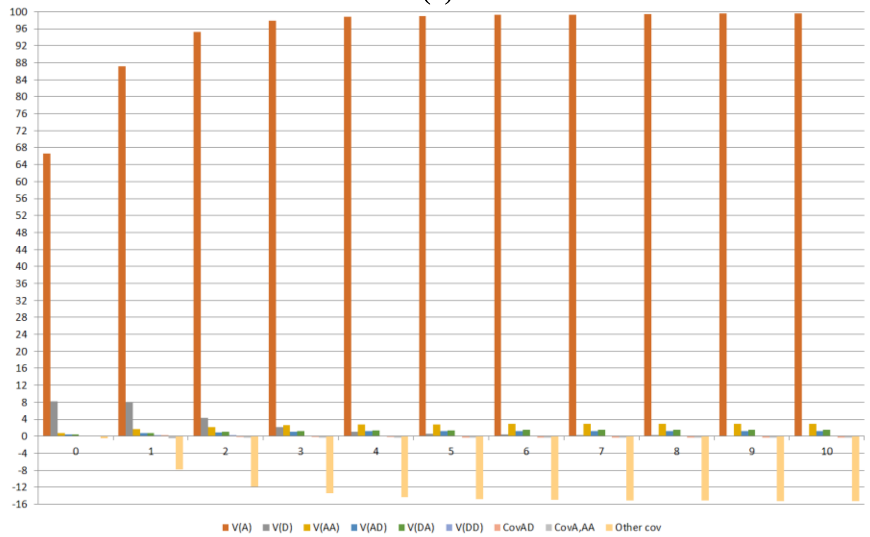

(d)

Additional Figure 7. Components of the genotypic variance in a population with high LD level, along 10 generations of random crosses (a and c) or selfing (b and d), assuming duplicate genes with cumulative effects, 100 (a and b) and 30% (c and d) of epistatic genes, and sample size of 5,000 per generation.

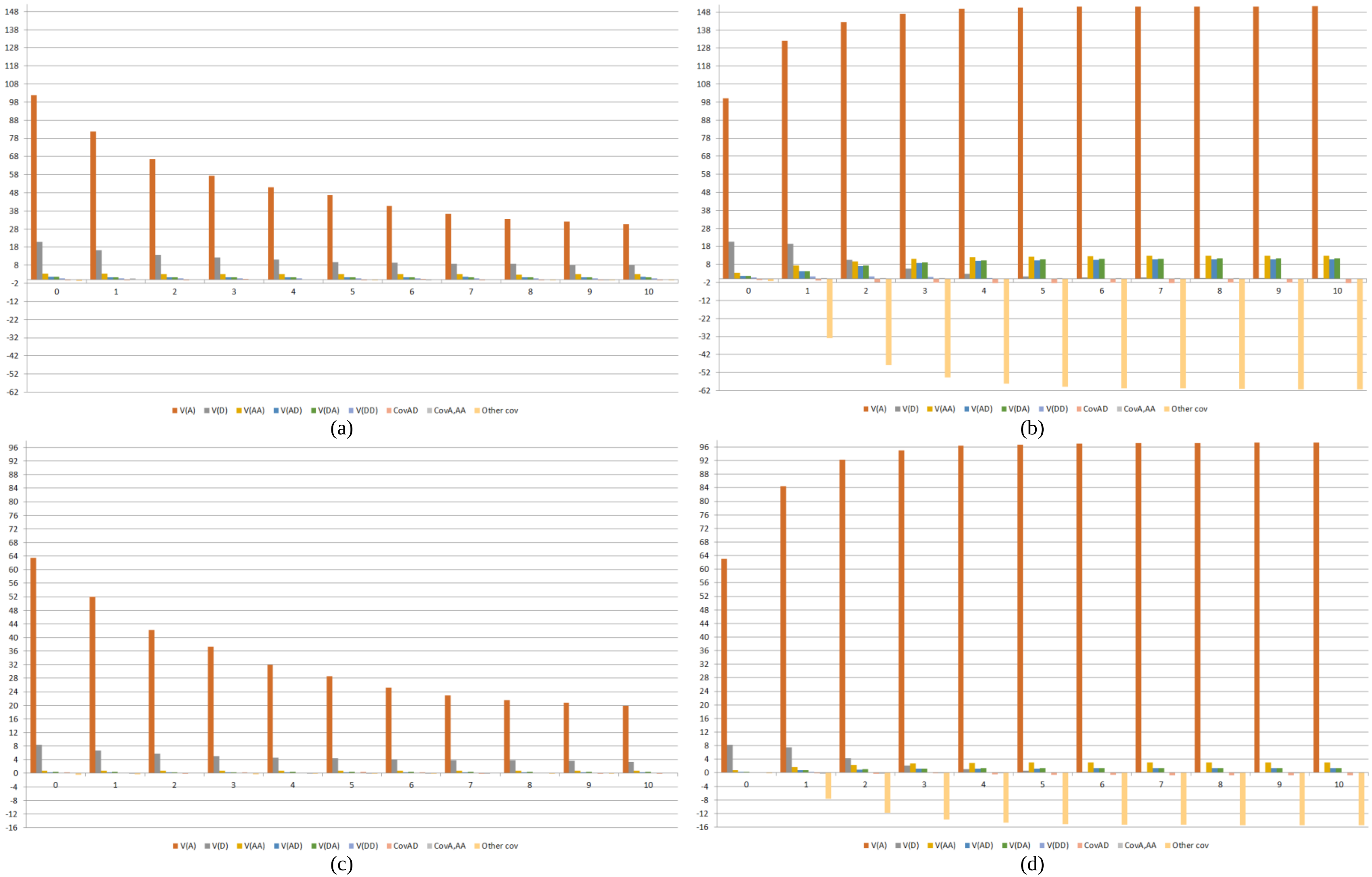

Additional Figure 8. Components of the genotypic variance in a population with high LD level, along 10 generations of random crosses (a and c) or selfing (b and d), assuming nonepistatic genic interaction, 100 (a and b) and 30% (c and d) of epistatic genes, and sample size of 5,000 per generation.

Appendix

For two genes, the genotype probabilities in generation 0 ($f_{ij}^{(0)}$) are presented by JMS Viana [24], where i and j (i, j = 0, 1, or 2) are the number of copies of the gene that increase the trait expression (A and B). For example, $f_{22}^{(0)} = p_a^2 p_b^2 + 2p_a p_b \Delta_{ab}^{(-1)} + \left[\Delta_{ab}^{(-1)}\right]^2$. After n generations of selfing, the genotype probabilities are:

$$f_{22}^{(n)} = f_{22}^{(0)} + (F/2)\left[f_{21}^{(0)} + f_{12}^{(0)}\right] + P_1^{(n)}$$

$$f_{21}^{(n)} = (1-F)\left[f_{21}^{(0)} + (1-c^n)f_{11}^{(0)}/2\right]$$

$$f_{20}^{(n)} = f_{20}^{(0)} + (F/2)\left[f_{21}^{(0)} + f_{10}^{(0)}\right] + P_2^{(n)}$$

$$f_{12}^{(n)} = (1-F)\left[f_{12}^{(0)} + (1-c^n)f_{11}^{(0)}/2\right]$$

$$f_{11}^{(n)} = (1-F)c^n f_{11}^{(0)}$$

$$f_{10}^{(n)} = (1-F)\left[f_{10}^{(0)} + (1-c^n)f_{11}^{(0)}/2\right]$$

$$f_{02}^{(n)} = f_{02}^{(0)} + (F/2)\left[f_{01}^{(0)} + f_{12}^{(0)}\right] + P_2^{(n)}$$

$$f_{01}^{(n)} = (1-F)\left[f_{01}^{(0)} + (1-c^n)f_{11}^{(0)}/2\right]$$

$$f_{00}^{(n)} = f_{00}^{(0)} + (F/2)\left[f_{01}^{(0)} + f_{10}^{(0)}\right] + P_1^{(n)}$$

where

$$P_1^{(n)} = (1/4)\left\{[F - (1-F)(1-c^n)]f_{11}^{(0)} + c_1(1-2r_{ab})\Delta_{ab}^{(-1)}\right\}$$

$$P_2^{(n)} = (1/4)\left\{[F - (1-F)(1-c^n)]f_{11}^{(0)} - c_1(1-2r_{ab})\Delta_{ab}^{(-1)}\right\}$$

$$c = 1 - 2r_{ab}(1-r_{ab})$$

$$c_1 = 2\{1 - [(1-2r_{ab})/2]^n\}/(1+2r_{ab})$$

and r_{ab} is the recombination frequency.
